# Extending conceptual frameworks: life course epidemiology for the study of back pain

**DOI:** 10.1186/1471-2474-11-23

**Published:** 2010-02-02

**Authors:** Kate M Dunn

**Affiliations:** 1 Arthritis Research Campaign National Primary Care Centre, Primary Care Sciences, Keele University, Keele, Staffordshire, ST5 5BG, UK

## Abstract

**Background:**

Epidemiological studies have identified important causal and prognostic factors for back pain, but these frequently only identify a proportion of the variance, and new factors add little to these models. Recently, interest has increased in studying diseases over the life course, stimulated by the 1997 book by Kuh and Ben-Shlomo, a move accompanied by important conceptual and methodological developments. This has resulted in improvements in the understanding of other conditions like cardiovascular and respiratory disease. This paper aims to examine how conceptual frameworks from life course epidemiology could enhance back pain research.

**Discussion:**

Life course concepts can be divided into three categories. Concept 1: patterns over time, risk chains and accumulation. Simple 'chains of risk' have been studied - e.g. depression leading to back pain - but studies involving more risk factors in the chain are infrequent. Also, we have not examined how risk accumulation influences outcome, e.g. whether multiple episodes or duration of depression, throughout the life course, better predicts back pain. One-year back pain trajectories have been described, and show advantages for studying back pain, but there are few descriptions of longer-term patterns with associated transitions and turning points. Concept 2: influences and determinants of pathways. Analyses in back pain studies commonly adjust associations for potential confounders, but specific analysis of factors modifying risk, or related to the resilience or susceptibility to back pain, are rarely studied. Concept 3: timing of risk. Studies of critical or sensitive periods - crucial times of life which influence health later in life - are scarce in back pain research. Such analyses could help identify factors that influence the experience of pain throughout the life course.

**Summary:**

Back pain researchers could usefully develop hypotheses and models of how risks from different stages of life might interact and influence the onset, persistence and prognosis of back pain throughout the life course. Adoption of concepts and methods from life course epidemiology could facilitate this.

## Background

Studies of back pain were historically based in the biomedical model of disease and illness, where symptoms were assumed to signify underlying diseases with known aetiology (e.g. injury caused by lifting), clear-cut pathology (e.g. prolapsed disc) and specific treatment (e.g. surgery). Many researchers have, more recently, embraced the biopsychosocial model, first described 30 years ago by Engel [1], which emphasizes the inter-relationships between biological, psychological and social factors [2]. Clinicians have also broadly embraced this conceptual shift; the biopsychosocial model is now incorporated into management [3], and treatment guidelines suggest that factors from all three domains should be addressed [4].

A significant body of research has developed in back pain with many studies focusing on biopsychosocial factors which inform the onset of back pain (e.g. [5-7]) and the likely prognosis of patients consulting with back pain (e.g. [8-11]). The World Health Organisation's International Classification of Functioning, Disability and Health [12] also influences research in back pain, with studies discriminating between measures of participation, such as work, and measures of impairments in bodily functions and structures, such as pain intensity. However, although many studies are increasingly including a wide range of appropriately measured factors, and being carried out prospectively, relatively few studies (e.g. [13-16]) have included follow-up beyond the 1-year period. As back pain is a long-lasting condition, that is present at all ages, studies covering a single year can only provide a snapshot of the whole picture, and may be missing vital information about the longer-term course.

Many studies begin by constructing statistical models of the association between a range of factors and outcome, but these often only account for a relatively small proportion of the total variance [17]. Surprisingly few studies include prior conceptual models of what they are hoping to identify or planning to test. Exceptions to this are models used in studies of psychological factors, for example the self-regulatory model of illness [18], and Bandura's model of self-efficacy [19]. However, these are not commonly developed for or among back pain sufferers, can refer to wide classifications of chronic illness, and rarely explicitly describe or test temporal relationships. Attempts to elucidate antecedent factors, and patterns and mechanisms of change in wider populations over time have been limited. Perhaps, in addition to improving our studies of the factors already identified,[17] and increasing the time-frames covered by the studies, further conceptual developments are needed to really understand how, when and why back pain occurs and recurs throughout an individuals' lifetime.

Over recent years, there has been increasing epidemiological interest in studying diseases over the life course, stimulated by the book by Diana Kuh and Yoav Ben Shlomo in 1997[20]. This move has been accompanied by crucial conceptual and methodological developments. Life course epidemiology is defined as "the study of long term effects on later health or disease risk of physical or social exposures during gestation, childhood, adolescence, young adulthood and later adult life"[21]. The approach can be used to develop conceptual models hypothesising causal links and pathways between factors occurring throughout the life course, and both the onset and progression of health or disease in adult life. In diabetes, cardiovascular and respiratory disease in particular, the application of principles now known as life course epidemiology have moved the focus of research from adult risk factors to consideration of markers of prenatal and infant growth. Barker et al. generated the foetal origins hypothesis [22], and proposed that foetal undernutrition is related to abnormal structure and function, and conditions such as coronary heart disease in adult life. This hypothesis has triggered hundreds of research studies, and has improved understanding of illness processes in cardiovascular disease. Debates on changes in policy as a result of these studies, such as interventions to reduce the frequency of low birth weight babies, have been stimulated [23,24]. Findings from these studies have also triggered the World Health Organisation to consider the key issues and implications for policy and research arising through life course perspectives[25].

Incorporating some of the fundamental concepts and models described within life course epidemiology into studies of back pain, with the biopsychosocial framework already embedded there, may substantially improve our understanding of the condition, and our ability to influence its course. This paper will focus on the application of concepts and models from life course epidemiology to the study of back pain, and highlight some of the advantages and limitations this poses. In this context, the term 'risk' will be used to refer both to risks of developing the disease, and risks of the disease persisting or worsening.

## Discussion

The life course approach offers a framework for research into the risks, onset and progression of health and disease based on biological, behavioural, psychological and social influences. A primary principle is that factors causing or influencing health and disease may occur during all stages of life: gestation, childhood, adolescence, in adult life or across generations[20].

### Fundamental concepts of life course epidemiology

Kuh and colleagues [26] refer to different groups of concepts in life course epidemiology. They describe types of pathways of causality over time, the mechanisms that influence or determine those pathways, and also specific timing of particular causal factors. The following is a summary of terms and concepts described by Kuh et al. [26] and Ben-Shlomo & Kuh [27].

#### Patterns over time, risk chains and accumulation

Patterns over time can be thought of in terms of patterns in single factors over time, or patterns among different factors (concept 1). 'Trajectories' describe patterns of single factors, e.g. trajectories of functional decline among cancer patients [28]. These may additionally be described with short-term changes, or 'transitions', and marked changes of directions, or 'turning points'. Patterns among different factors may occur sequentially in 'chains of risk', or may be co-occurring, either independently or clustered (e.g. a range of factors linked to a family or household), and lead to 'accumulation of risk'. All of these types of change over time or causal pathways may combine elements from biomedical, psychological or social domains.

#### Influences and determinants of pathways

These pathways may be influenced or determined by other processes (concept 2). These include effect modification, where a causal pathway actually differs across levels of a modifying factor (e.g. the relationship between coffee consumption and coronary calcification may differ between men and women [29]), and mediation, where a factor on the causal pathway (i.e. after the risk) mediates an association. Other mechanisms include resilience and susceptibility, which are dynamic and potentially modifiable processes that can lead to either reduced or increased risk of disease at particular times; for example, a strong sense of coherence may improve resilience to cancer[30]. These influencing factors may be at the individual level, which are the most common factors studied, familial factors such as marital functioning [31] or may be area level factors, which have been identified as being often neglected in chronic pain research[32].

#### Timing of risk

The third conceptual area surrounds the timing of risk. Critical period models refer to crucial times in development where a risk factor can influence health later in life, for example lower socioeconomic status at the ages of 2-3 years may increase risk of respiratory diseases[33]. Sensitive periods are similar, but the excess risk can also occur outside the sensitive period, albeit at a reduced strength. Induction and latency periods are more commonly used terms, and refer to the time between exposure and disease initiation, or between disease initiation and detection, respectively. Birth cohort effects are also important, and relate to differences between groups of people depending on when they were born; for example, people born in the 10 years following 1945 had poorer self-rated health, which declined more rapidly, than people born in the preceding 10 years[34].

These concepts are not all new, and are not fixed. This is a developing area, and there is scope for additions and re-definitions as ideas and research develops. Methods used in life course epidemiology are developing, and include techniques such as latent class analysis (e.g. [35,38]), generalised estimating equations (see Twisk et al. [39]), life table analysis (e.g. [40]), reduced rank regression (RRR) analysis (e.g. [41]) and K means analysis (see Brossart et al. [42]); for discussion of these and other methods (see Pickles et al. [43,44]. and Elder & Giele [45]).

### Specific application to back pain

**Figure 1:**
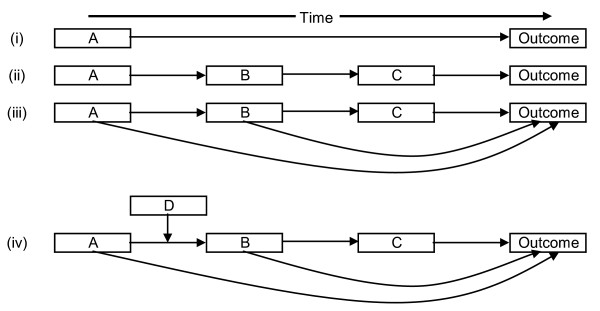
**Illustration of chains of risk**. (*Figure adapted from Kuh et al*. [20])

The most common type of prospective aetiological study in back pain is an investigation of how variables collected at time one lead to an outcome at time two. This may be how factors such as depression or workload at initial data collection are risk factors for the onset of back pain at follow-up (e.g. [5,46]), or how factors such as pain levels or psychological distress around the time of consultation predict poorer prognosis at follow-up (e.g. [47,48]). These studies commonly adjust for potential confounders, indicating the independence of the identified factors. Such studies may be conceptualised as examining chains of risk, although the chains involved usually only have one risk factor in the chain (see Figure 1, study type (i)). They may also represent studies of simple transitions from pain free to back pain, or from mild pain to severe pain; risk and outcome are often not clearly distinguished, for example the level of disability at one stage may be a key predictor of the level of disability at the next. Such risk chains may be oversimplifications of the true situation, and examples of increasingly complex risk chains are shown in Figure 1.

Epidemiological studies of back pain which involve more than one risk factor in the chain are rare or non-existent, possibly due to the relatively short term nature of most back pain studies (i.e. one year), the lack of repeated measurements beyond baseline and follow-up, and the difficulties in conducting longer studies, but also due to the lack of underlying hypothesized causal pathways the testing of which would necessitate longer studies. Figure 1 (ii) shows an example of such a pathway, where A leads to B, which leads to C, which links with the outcome. For back pain, an example might be a study of healthcare consulters, where pain (A), leads to problems sleeping (B), which leads to depressive symptoms (C), which predicts outcome (persistence of back pain). This may be a simplification for most circumstances, and Figure 1 (iii) shows how A and B may also lead to outcome, independently of the direct chain. In turn, there may be mediators of the associations shown in (ii) and (iii), as illustrated in Figure 1 (iv), where factor D influences the link between A and B; in our example this might be how effective pain management (D) could reduce the likelihood of pain leading to sleep problems.

Chains such as these may also be 'protective', thereby reducing the likelihood of a poor outcome. An example of this among back pain sufferers might be where good social support (A), leads to positive beliefs about back pain (B), leading to active coping strategies (C), and therefore reduced likelihood of pain persistence (outcome). These examples are hypothetical, and construction of such models would require knowledge of all elements of the chain, and prior hypotheses about how such chains could occur. Such chains would also require empirical testing before indicating their causality.

Most studies identifying factors such as seen in Figure 1 (i) include a range of different factors, and combinations of these may lead to accumulation of risk; this may be accumulation over time, or accumulation across different risk factors. Similar diagrams, such as directed acyclic graphs [49,50] can show how causal inference can be estimated, and can facilitate the study of acute exacerbations of chronic diseases[51]. Multivariate analyses are useful in terms of prediction, and studies producing clinical prediction rules (e.g. [52,53]) indicate how these can provide valuable information in determining response to treatment or return to work. Other work combining 'risk' information is also useful in determining likelihood of pain persistence or chronic pain (e.g. [54-56]). However, collection of this data at a single time point, with little or no interpretation of whether there was any sequence to the development of the risk factors, substantially limits our understanding. Some risk factors may accumulate over time, for example depression measured at one time point has been shown to predict both back pain onset [57] and persistence [58], but cumulative depression over the life course may be more important. In addition, other factors may modify the relationship between depression and back pain in important ways; such effect modifications should be modelled prior to the main analyses, and should be identified and interpreted during analysis, not simply adjusted for.

**Figure 2:**
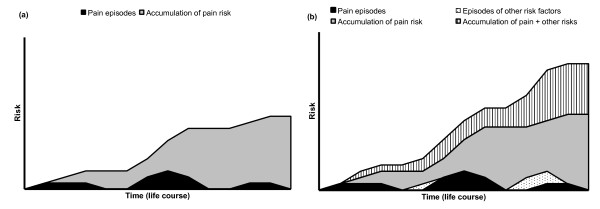
**Graph showing accumulation of risk**.

Traditionally in back pain research, each episode of back pain has been treated as a separate entity, with history of back pain (identified at baseline) treated as a risk factor for incident back pain (e.g. [59]), or an indicator of prognosis (e.g. [47]). This approach has probably been taken for historical reasons. For example, many early studies in back pain included participants from insured or worker's compensation schemes, where episode inception was (for administrative purposes at least) a back injury, often work-related. This appears to ignore the likely natural history of back pain as a syndrome. The very fact that prior pain experience influences the future course should indicate that back pain is experienced over the life course, and therefore needs to be studied in the longer term. It seems likely that there is an accumulation of risk over time for the pain itself [See figure 2a]. This is supported by studies showing that increasing duration of episode at study baseline is a strong predictor of both persistence [60] and poorer response to treatment [61]. Recent work on trajectories of back pain [36] gave short term (one year) views of how back pain itself might change over time; the participants with the more severe trajectories also had longer pain duration, indicating an accumulation of pain risk. However, they also had increased levels of other factors such as other pains and psychological problems, indicating that the accumulation of risk is not isolated to the pain, but covers complex factors across the biopsychosocial framework [See figure 2a]. This accumulation of wider risk is similar to amplification in the model of back pain staging by Raspe et al. [62], but at present this is a model of hypothetical stages modelled on classification of groups of people at a point in time, rather than evidenced by observed progression from one stage to the next. One argument against the idea of accumulation of risk for back pain over the life course is highlighted by examining data on the prevalence of back pain at different ages. Studies in children indicate a one-year prevalence of back pain of 18-26% [63,64], studies among adults (predominantly working age) indicate prevalence of between 36% and 72% [65], and studies in older people show a one-year prevalence of between 21% and 32% [66,67], with back pain still present in centenarians [68]. So back pain is present throughout the life course, and does not appear to increase linearly with age as would be expected with a simple accumulation of risk over time, although a review specifically examining this reported that an increase with age was more apparent if only severe cases were included [69]. Longer term studies may begin to disentangle apparent contradictions, identify patterns across the whole life course, both of pain and of its related influences, and provide information about life course trajectories with associated transitional factors and turning points.

The idea that both back pain itself, and many of the other risk factors for it, can be episodic, recurrent or persistent means that the likelihood of chains of risk moving neatly from cause to effect is improbable. It is possible that the pain and the risk factors may occur in series (e.g. an episode of depression precedes an exacerbation of back pain), or may fluctuate in parallel (e.g. episodes of back pain and depression occur at the same time). These types of problems are likely to need sophisticated theoretical modelling and statistical analysis to investigate fully.

The concepts of life course epidemiology were defined subsequent to Barker's studies of the influence of birth weight on cardiovascular disease, and a large number of papers identifying themselves as life course studies investigate factors in gestation or early childhood, conceptualised as 'critical periods'. There are few of these types of studies looking at back pain, or musculoskeletal conditions in general. One study using the 1958 British Birth Cohort showed an association between social class at birth and back pain at age 33 among women, but not men [70]. A study in Norway found an association between parental education measured early in the subject's life and musculoskeletal sickness absence in their 20s and 30s [71]. One group has linked birth weight and neonatal hospital admission, and pain in the individual or their family or friends during childhood, with having chronic pain as a young adult[72,73]. Studies that are likely to be related to back pain include one showing that early life exposure to tobacco smoke was related to having long-term sick leave as an adult [74], and another showing that factors such as birth weight and maternal marital status were related to disability pensioning in adult life [75]. Studies of unexplained symptoms (including back pain) have shown that parental ill-health (particularly of the father) in childhood is associated with the presence of multiple symptoms in adulthood, but severe childhood physical disease in the individual was not associated with adult symptoms [76,77].

Modelling and testing of clear pathways showing how these factors might relate would be useful for future studies; they may be related to critical periods of biological development, they may form part of chains of risk as yet not fully described, or they may be markers for other related factors. One factor that may be an element in these pathways is cortisol, which has been linked with gestational factors [78,79] and with chronic pain [80]. Muscle strength has also been linked with birth weight and pain [81,82], although it's role in the development and persistence of back pain is inconclusive [83,84]. Genetic influences have been identified for back pain [85,86], disc disease [87], and disc degeneration [88], although the mechanisms for these links require further study. Smith et al. [89] highlight a number of additional biological risk factors and mechanisms that could provide new knowledge about chronic pain. Other pathways linking early life factors with back pain remain to be described, but appropriate models are likely to be complex and to involve factors from biological, psychological and social domains. One challenge will be identifying factors to be included in such pathways. These could come from conceptual models, possibly developed based on knowledge from aetiological and prognostic studies, as well-designed observational studies can provide valid information on potential causal factors[90]. Potential factors could also be identified through communication between researchers from different fields (basic science, biomechanics, psychology, sociology, epidemiology etc.).

It should be remembered that life course epidemiology is not limited to early life periods, and as the definition indicates, factors identified or occurring in adolescence or adulthood are also of importance in studying a condition with a life course approach. However, evidence from other conditions such as cardiovascular diseases indicates that research should not be limited to factors relating to adulthood or occurring around the same time as the condition. Back pain researchers should develop hypotheses and models of how risks from different stages of life might interact and influence the onset, persistence and prognosis of back pain.

### Theoretical life course model for back pain

**Figure 3:**
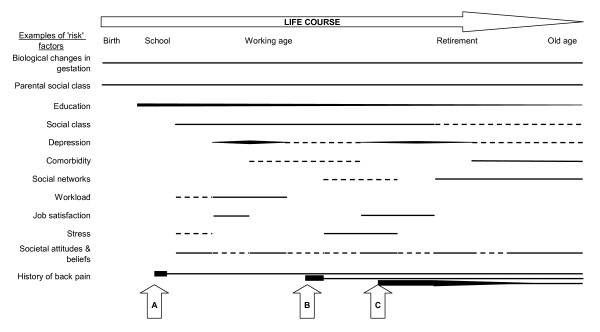
**A conceptual example of back pain risk over the life course**.

Back pain appears to have a complex risk framework; some risk factors may potentially be determined during gestation or early childhood and remain throughout the life course, others may be determined later in life, but also remain throughout, others may be more intermittent, appearing or disappearing at certain points, and being of different strengths at different times. Figure 3 shows a hypothetical model of how risk might develop for an individual over their lifetime, incorporating risk factors from different domains of the biopsychosocial model. A traditional approach to assessing risk (represented here by the black horizontal lines) at a particular time might be to take a vertical 'snapshot' of risk density. Taking into account accumulation of risk, a person's risk at a particular time might be better assessed by looking back from that point at their whole life experience. Certain risk factors may be more important when they first occur, represented by a broader line at inception, which narrows over time. Other factors may build up and reduce during an 'episode', represented by broadening and narrowing of the lines. Some may be less important at certain times, represented in Figure 3 by dashed lines.

For the hypothetical person represented in Figure 3, there may have been biological changes during gestation, and parents in lower socioeconomic groups, that put them at increased risk of back pain episodes or persistent back pain during their lifetime. They may then have had restricted educational opportunities, which is a risk factor that remains throughout their lives, but diminishes in importance over time. They may have been in a lower socioeconomic grouping themselves, which influences their risk of back pain throughout working life, but perhaps less so during retirement (represented by dashed line). They may experience intermittent periods of depression, which increase their risk at the time, more so at certain points. They may have some comorbid illness during adulthood which slightly increases their risk, but more serious comorbidity in older age, which increases their risk more. Carrying a heavy school bag may have been a workload that slightly increased their risk as a child, but the workload associated with their job increased their risk more during their early working life, although this risk disappeared when they got promoted to a job with lighter duties. Throughout their life they may have periods where they have poor job satisfaction or high levels of stress, which might increase their risk at that time. They might also be influenced by societal attitudes and beliefs about back pain, perhaps differently at different times. They also had an episode of back pain at school (point A), and one in their early working life (point B), which both resolved fairly quickly (as they had relatively few risk factors), but left them with an increased residual risk of future episodes. They then have another episode of back pain at point C, when they are also depressed and dissatisfied with their job, and this, combined with the lifelong risk factors, and the previous history, means that this episode persists and becomes chronic pain.

This model is purely hypothetical, but could be reproduced using empirical data, for individuals or groups of people, to estimate their risks at various life stages. Factors such as life events could be added, as could treatments and protective factors such as self-care activities. It should be recognised that even the influence of risk factors that have already occurred is not fixed, for example increased vulnerability to poor coping with job difficulties resulting from their parents socio-economic status might not be a problem with their job during one period of their life, but might become a problem, leading to back pain, if they move into a different work situation. It is therefore important to recognise the setting or context of risk factors when assessing their impact. What this model does not present is causal pathways; these might be more simply presented in the types of diagrams in Figure 1. Figure 3 adds to the current model of assessing risk, which commonly only identify factors present at that time, thereby missing or ignoring factors that occurred in the past but have a residual effect. This model could be expanded to incorporate new risk factors and causal pathways as they are identified, and it could be used to model both risk of onset and prognosis, particularly if the risk factors are the same. It could also be used to investigate issues such as whether there is a threshold level of risk density at which people develop long-term back pain (thereby facilitating intervention before a critical threshold is reached), or explain why some people seek healthcare while others do not, although their experience of pain may be similar.

In the long-term, further understanding of back pain stimulated by using life course methods could potentially have clinical and policy implications. For example, if there is accumulation of risk over time for the back pain itself, intensively managing the first or early episode of back pain that a person experiences might have long term benefits in terms of prevention of later episodes and potential chronicity. This might mean intervening on back pain among children or adolescents in a more intensive or different manner, e.g. recognising and managing their pain more effectively, or providing them with improved education on how to manage their symptoms. Where causal pathways are elucidated, intervening to prevent progression of the condition might be more appropriate, for example as mentioned above, treating sleep problems in newer episodes of back pain may prevent progression of the pain itself, and prevention of depression. However these examples are hypothetical, and before any policy implications can be understood, further research is needed.

### Anticipated advantages and difficulties

Shifting the conceptual basis of the study of a condition is not a simple process, and there are factors about back pain that will make it more difficult, but also factors that are facilitative. The prior adoption of the biopsychosocial model within the field of back pain is a distinct advantage, and in some ways could allow for a broader expansion of the concepts than some other conditions have achieved. Knowledge of the occurrence and associations with back pain is also beneficial, as there is a broad basis on which to build.

When applying a life course epidemiological approach to chronic oral conditions, Nicolau and colleagues [91] highlighted five characteristics that make a condition appropriate to study with the life course approach. Their first point was that a chronic condition is easier to study as it can be identified more simply. While back pain can be thought of as a chronic condition in many circumstances, it is also inherently episodic and recurrent and therefore may be difficult to detect at certain stages. People attempt to overcome this by collecting information about history of the condition, but recall is inevitably imperfect, and errors are created. Compiling a more complete natural history of back pain across the life course would be helpful, and perhaps improvements in the collection of history might help, beyond whether someone has experienced back pain in the recent past, or in their lifetime.

The second point made by Nicolau and colleagues [91]is that a condition which is cumulative would be advantageous, as the degree of disease development could be identified. This is difficult for back pain. Traditionally distinction was made between acute and chronic cases based on time since onset, but this does not seem appropriate if acute cases with a history of the problem have a different prognosis, and it has been highlighted that there are differing degrees of chronic back pain. Some authors have attempted to describe phases in the course of back pain; these range from the biomedical 'ageing' processes described by Laurens Rowe in 1983 [92], to the widely used Chronic Pain Grades characterized by Von Korff et al. [93], the stages of back pain described by Raspe et al. [62], and more recently an ordering of back pain trajectories suggested by Dunn et al. [36]. If we are to accept that back pain is a life-long process, or a condition experienced in the long-term, we need to build on this work to really gain a deeper understanding of the development and progression of back pain over time. These may be conceptualised using life-course epidemiological terminology as trajectories, which may be characterized by transitions and turning points.

The third point identified was that the condition should be measurable in a valid and reliable manner. Back pain is predominantly a symptom-based condition, without clear diagnosis, and this therefore poses challenges for study using a life course approach. Progress has been made on this in back pain, there are instruments that have been shown to be valid and reliable (e.g. [94-97]). However, there is the issue of what to measure. Do we measure the pain? Or the associated disability? Perhaps range of motion? Or measures of participation such as return to work? The recommendation is to assess a range of measures [98,99,17], with the primary measure dependent on the setting. Recommendations have also been published for measuring the prevalence of back pain, and using these to provide consistent and reproducible estimates is likely to be beneficial [100].

The fourth and fifth points made by Thomson et al. are much more manageable for back pain. They say that the condition should be prevalent so that sample sizes are achievable - with prevalence figures at a general minimum of 20% this should not be a problem. They also say that the condition being studied should have public health relevance, and with the high prevalence, the considerable use of healthcare and substantial costs of work loss associated with back pain [101,102], this should not be disputed.

Another advantage of studying back pain within a life course approach is that it is commonly measured within cohort studies looking at general health. Studies such as the UK Millennium Cohort Study [103] and the 1958 and 1970 British Birth Cohorts [104,105] include measures of back pain, and many other long-term cohorts are also likely to contain relevant information. These are resources currently under-utilized by back pain researchers. However, using such studies comes with methodological challenges, such as the potentially inadequate measurement of back pain when compared with more focused studies [106]. One alternative approach is to combine data from current surveys with historical administrative data, such as birth records as used in Mallen et al[72]. This is likely to be a problem if back pain severity or phase is to be studied, and researchers may have to be innovative in their methods and analyses, cautious in their interpretations, or persuasive in their arguments to researchers following up the cohorts to include improved measures of back pain.

There are also practical limitations to using life course epidemiological methods. These include the high costs and long follow-up times required to collect appropriate prospective data, and the reluctance of funding agencies to provide support for such long term and intensive studies. Incorporating questions into ongoing longitudinal studies, as mentioned above, is one solution, but if the advantages and strengths of such designs can be communicated to funding agencies, back pain specific studies may be possible. Another potential problem, inherent in most follow-up studies but particularly relevant for long-term studies, is attrition, which can affect the validity of data available, and limit the generalisability of study findings. Other potential limitations may become apparent as more researchers use life course methods in back pain research.

## Conclusions

Epidemiological research in back pain seems to, with notable exceptions, have set aside the basic epidemiological tenets of time, place and person, and focused far more on the 'person'. This may be because these other types of research are perceived as being too difficult to study or understand. Using a life course approach might give us the tools by which we can begin to approach these complex issues, and move on from where we are at present. It can be seen as a method of taking experiences, influences and problems from before birth, early life, adulthood and older age, from within the individual, and from their wider social, cultural, economic and occupational setting into account when investigating back pain.

Thinking about the life course approach in the context of the study and understanding of back pain raises a number of questions that cannot be answered at present. For example, are there critical periods in gestation or early life development that influence back pain (concept 3)? And if so, do they influence the first onset of the condition, or the development of chronicity, or both? And by what mechanisms might these occur? Do any factors mediate these patterns (concept 2)? We may be able to apply some of the concepts and methods from life-course epidemiology in order to begin to answer these and numerous other questions.

Concept 1 highlighted the study of trajectories, and these are generally poorly understood in terms of the natural history of back pain. We need to determine whether back pain is a condition characterized by recurrent but unrelated episodes, or whether it is a condition that can progress through phases. Is the propensity for chronic back pain a trait which is set early in life, or is it a state experienced according to the situation at the time? Better understanding of longer-term trajectories of back pain will improve our understanding, and enable testing of potential models of back pain progression and improve our knowledge of back pain trajectories across the life course.

We also need to think about whether there are chains of risk or an accumulation of risk for back pain, and again, whether this is risk of onset or persistence, or both. While very specific pathways, such as modelling psychological chains of risk, or determining social influences, are important, broader models linking elements from bio- psycho- and social domains are likely to be the most productive in really gaining a deeper understanding of the problem. In the theoretical life course model for back pain presented here, factors from all these domains could be incorporated to get an idea of risk accumulation over time and across factors. There will be a balance to be made between developing sophisticated models and collecting data on myriad factors, and actually designing studies that are feasible and are likely to have interpretable findings. Identifying feasible, but relatively simple, chains of risk, as well as the factors modifying or mediating those chains, will be most useful.

In this paper, the life course approach is described in the context of back pain. However, many of the concepts, theories, arguments and principles are equally applicable to the study of other symptom-based conditions such as other non-specific regional and widespread pains, headache and irritable bowel syndrome. As many of these types of conditions share common epidemiological features [107] and are likely to overlap [108], it is possible that not all research needs to be replicated within each condition, and researchers may be able to 'borrow' research and ideas from studies of other and different symptoms.

We have used examples from prior research, and hypothetical studies, to illustrate the ways in which life course approach could be incorporated into the study of back pain, and highlighted advantages and difficulties of using this approach. It can be seen as a conceptual approach to facilitate the identification of specific testable hypotheses. Researchers will need to develop and test their own models using their knowledge of the field and hypotheses about the course of back pain. We all need to take the evidence we have accumulated, build on the methods and models we already use, and expand these, working with colleagues from other fields and borrowing from the epidemiological life course approach, to gain a deeper understanding of causal pathways and mechanisms of back pain over the life course. In time, it may be possible to adopt a common paradigm for back pain over the life course.

## Summary

1. Back pain research is limited by the focus on the adult with an episode of pain; principles from life course epidemiology may be used to change the focus of research.

2. New methods of studying back pain over time would be beneficial, including trajectories of pain and chains of risk.

3. More research needs to focus on factors modifying risk, or related to the resilience or susceptibility to back pain

4. Researchers should develop hypotheses and models of how risks from different stages of life might interact and influence the onset, persistence and prognosis of back pain.

## Authors' contributions

KMD carried out all the work in relation to this paper.

## Authors' information

KMD is an epidemiologist who holds a Wellcome Trust Research Career Development Fellowship of which one focus is the study of pain and symptoms over time. Some of the ideas for this paper were developed while on sabbatical at Group Health Center for Health Studies in Seattle, USA.

## Pre-publication history

The pre-publication history for this paper can be accessed here:

http://www.biomedcentral.com/1471-2474/11/23/prepub
